# Decreased oxygen extraction during cardiopulmonary exercise test in patients with chronic fatigue syndrome

**DOI:** 10.1186/1479-5876-12-20

**Published:** 2014-01-23

**Authors:** Ruud CW Vermeulen, Ineke WG Vermeulen van Eck

**Affiliations:** 1 CFS/ME Medical Centre Amsterdam, Waalstraat 25-31, Amsterdam 1078BR, Netherlands

**Keywords:** Chronic fatigue syndrome, Exercise test, Exercise intolerance, Oxygen extraction

## Abstract

**Background:**

The insufficient metabolic adaptation to exercise in Chronic Fatigue Syndrome (CFS) is still being debated and poorly understood.

**Methods:**

We analysed the cardiopulmonary exercise tests of CFS patients, idiopathic chronic fatigue (CFI) patients and healthy visitors. Continuous non-invasive measurement of the cardiac output by Nexfin^®^ (BMEYE B.V. Amsterdam, the Netherlands) was added to the cardiopulmonary exercise tests. The peak oxygen extraction by muscle cells and the increase of cardiac output relative to the increase of oxygen uptake (ΔQ’/ΔV’O_2_) were measured, calculated from the cardiac output and the oxygen uptake during incremental exercise.

**Results:**

The peak oxygen extraction by muscle cells was 10.83 ± 2.80 ml/100ml in 178 CFS women, 11.62 ± 2.90 ml/100 ml in 172 CFI, and 13.45 ± 2.72 ml/100 ml in 11 healthy women (ANOVA: *P*=0.001), 13.66 ± 3.31 ml/100 ml in 25 CFS men, 14.63 ± 4.38 ml/100 ml in 51 CFI, and 19.52 ± 6.53 ml/100 ml in 7 healthy men (ANOVA: *P*=0.008).

The ΔQ’/ΔV’O_2_ was > 6 L/L (normal ΔQ’/ΔV’O_2_ ≈ 5 L/L) in 70% of the patients and in 22% of the healthy group.

**Conclusion:**

Low oxygen uptake by muscle cells causes exercise intolerance in a majority of CFS patients, indicating insufficient metabolic adaptation to incremental exercise. The high increase of the cardiac output relative to the increase of oxygen uptake argues against deconditioning as a cause for physical impairment in these patients.

## Background

Exercise intolerance is a frequent complaint from patients who meet the criteria for Chronic Fatigue Syndrome/Myalgic Encephalitis (CFS) and Idiopathic Chronic Fatigue (CFI) [1].

Objective tests for physical impairment measure the maximal oxygen uptake (peak V’O_2_) during a cardiopulmonary exercise test (CPET) [2-4]. Most studies agree that peak V’O_2_ is lower in CFS, but we need to understand the cause of the lower peak V’O_2_ to explain the pathogenesis.

The V’O_2_ depends on the uptake, transport and metabolism of oxygen in the muscle cells during physical exercise. In most CPET studies in CFS patients, the limitation of peak V’O_2_ is not attributed to a lower uptake and transport of oxygen to the muscle. A lower metabolic capacity of the muscle cell would change the demand for oxygen and thus lower the oxygen extraction (C(a-v)O_2_) and increase the cardiac output relative to V’O_2_ (ΔQ’/ΔV’O_2_) [5,6]. In previous studies we, and others, did not find impaired mitochondrial activity to be a cause for a lower peak V’O_2_[4,7], but abnormal mitochondrial activity was reported by some in CFS and CFI [8-10].

The aim of the present retrospective study was to determine to what extent the physical impairment in CFS and CFI was attributable to changes in uptake, transport and metabolism of oxygen in the muscle cells.

## Methods

Data was collected from patients who attended the CFS Medical Centre Amsterdam. The data of sedentary men and women, physically active less than 1 hour per week, was added to the patient database. This group comprised visitors to the centre for cardiopulmonary exercise tests, check-ups and training program advice over the same period. We obtained information about the health status of this group, but laboratory tests were not included. Subjects using medication that could possibly influence the pulmonary, cardiovascular, immunologic system or cellular respiration were not included in the study. Chronic fatigue patients were assigned to the CFS or CFI group according to the criteria of Fukuda [1]. Operational criteria for assignment were: a score of >40 on the fatigue subscale of the Checklist Individual Strength (CIS-20) [11], score of?≤?35 for vitality, ≤ 62.5 for Social Functioning and ≤50 for Role-Physical on the SF-36 [12] and ≥4 positive scores (≥7,5) of the additional symptoms of the CDC Symptom Inventory –DLV [13] for the diagnosis of CFS and ≤4 positive scores (≥7,5) of the additional symptoms of the CDC Symptom Inventory –DLV for the diagnosis of idiopathic chronic fatigue (CFI) . The cognitive function of patients was screened with the Shifting Attentional Test Visual of the Amsterdam Neuropsychological Tasks [14]. Normal Z-score in this test is -2 to +2.

All patients completed a CPET as part of the diagnostic procedure. The protocol of the CPET was described before [4]. All subjects performed a symptom limited CPET on a cycle ergometer (Excalibur, Lode, Groningen, The Netherlands) as described by Wassermann et al. [15]: 3 min without activity, 3 min of unloaded pedalling, followed by cycling against increasing resistance until exhaustion (ramp protocol) and concluded by 3 min cycling without resistance. The work rate increase was estimated from history, physical examination, gender, weight and height. Verbal encouragement was used to maximise performance during the last phase of incremental exercise. Exhaustion of the leg muscles was the limiting symptom in all participants. The V’E, V’O2, V’CO_2_ and oxygen saturation were continuously measured (Metasoft). The ECG was continuously recorded and blood pressure was measured every 2 min. Maximal exercise capacity was expressed as peak oxygen uptake per kilogram bodyweight and as percentage of predicted maximal oxygen uptake [16]. Non-invasive stroke volume measurement by continuous beat-to-beat pulse contour analysis (Nexfin) was added to the standard measurements of the CPET [17]. Oxygen extraction and ΔQ’/ΔV’O_2_ were calculated from the oxygen uptake and the cardiac output (C(a-v)O_2_ = 100 × V’O_2_/ CO and (Q’max-Q’rest)/(V’O_2_max-V’O_2_rest)) [18]. All subjects signed an informed consent for the use of the data for analysis and publication.

Statistical analysis was conducted using the IBM Statistical Package for the Social Sciences (19.0 for Windows, Chicago, Ill, US). The results were presented as the mean?±?standard deviation (SD). Kolmogorov-Smirnov tests with Lilliefors significance correction for normality showed that the data were normally distributed. Differences between groups were tested with Analysis of Variance (ANOVA) and Bonferroni post hoc analysis. Pearson product-moment correlation coefficient was calculated as a measure of the strength of the relationship between 2 variables. Statistical significance of two-tailed tests was determined by an alpha level of 0.05.

## Results

Data was collected and analysed from a total of 444 subjects who visited the CFS/ME Medical Centre Amsterdam between June 2008 and June 2013: 203 CFS patients (178 women), 223 CFI patients (172 women) and 18 healthy visitors (11 women) (Table 1).

**Table 1:** Demographic data and results

**Female**					
		**CFS**	**CFI**	**Healthy**	**ANOVA**
		**n=178**	**n=172**	**n=11**	***P***
Age	(years)	37.3 ± 12.1	37.9 ± 12.3	42.2 ± 14.8	0.421
Weight	(kg)	69.5 ± 15.0	67.5 ± 13.1	68.6 ± 13.2	0.412
BSA	(m^2^)	1.81 ± 0.20	1.78 ± 0.18	1.79 ± 0.17	0.497
Haemoglobin	(mmol/l)	8.45 ± 0.55	8.46 ± 0.51		0.846
Rest					
HR	(beats/min)	96.1 ± 14.9	93.5 ± 16.8	87.7 ± 11.1	0.113
V’O_2_	(ml/min/kg)	5.46 ± 0.99	5.41 ± 0.93	5.18 ± 0.98	0.635
O_2_ pulse	(ml/beat)	4.00 ± 0.85	3.95 ±0.82	4.09 ± 0.90	0.784
RER		0.86 ± 0.07	0.86 ± 0.07	0.81 ± 0.03	0.073
V’E/V’CO_2_		34.2 ± 4.3	34.3 ± 4.1	34.7 ± 3.0	0.924
SVI	(ml/m^2^)	42.8 ±7.0	42.3 ±7.6	43.4 ± 10.4	0.797
Cardiac index	(L/min/m^2^)	4.10 ± .081	3.97 ± 0.92	3.80 ± 0.95	0.294
O_2_ extraction	(ml/100ml)	6.48 ± 1.65	6.58 ± 1.72	6.96 ± 1.63	0.620
Anaerobic threshold					
HR	(beats/min)	112.0 ± 15.6	112.3 ± 15.2	113.3 ± 8.7	0.955
V’O_2_	(ml/min/kg)	10.9 ± 2.6	11.6 ± 2.7	13.7 ± 3.6	0.001
O_2_ pulse	(ml/beat)	6.75 ± 1.63	6.95 ± 1.67	8.33 ± 2.31	0.009
RER		0.84 ± 0.07	0.85 ± 0.08	0.81 ± 0.06	0.216
V’E/V’CO_2_		29.7 ± 3.5	28.8 ± 3.5	27.6 ± 1.7	0.025
SVI	(ml/m^2^)	45.7 ± 7.9	45.0 ± 8.2	48.2 ± 11.8	0.382
Cardiac index	(L/min/m^2^)	5.12 ± 1.10	5.11 ± 1.21	5.52 ± 1.64	0.534
O_2_ extraction	(ml/100ml)	8.30 ± 2.08	8.77 ± 2.23	9.86 ± 2.37	0.019
Peak exercise					
HR	(beats/min)	158.4 ± 19.3	159.6 ± 18.6	164.1 ± 11.3	0.250
V’O_2_	(ml/min/kg)	20.3 ± 5.0	22.2 ± 5.3	27.4 ± 7.2	0.000
V’O_2_/pred.	%	80.6 ± 17.9	83.1 ± 18.0	105.4 ± 18.8	0.000
O_2_ pulse	(ml/beat)	8.89 ± 2.17	9.26 ± 2.08	11.36 ± 2.89	0.001
RER		1.16 ± 0.10	1.18 ± 0.10	1.20 ± 0.11	0.094
V’E/V’CO_2_		30.2 ± 3.7	29.7 ± 3.7	27.9 ± 2.4	0.103
SVI	(ml/m^2^)	45.9 ±7.9	45.4 ± 8.3	47.6 ± 11.2	0.797
Cardiac index	(L/min/m^2^)	7.33 ± 1.77	7.39 ± 1.78	7.87 ± 1.96	0.618
O_2_ extraction	(ml/100ml)	10.83 ± 2.80	11.62 ± 2.90	13.45 ± 2.72	0.001
Slope					
ΔQ’/ΔV’O_2_	(L/L)	7.35 ± 2.40	6.80 ± 1.99	5.85 ± 1.23	0.012
**Male**					
		**CFS**	**CFI**	**Healthy**	**ANOVA**
		**n=25**	**n=51**	**n=7**	***P***
Age	(years)	41.6 ± 12.3	41.8 ± 9.9	49.4 ± 13.8	0.215
Weight	(kg)	88.9 ± 15.7	80.0 ± 12.8	94.3 ± 13.9	0.005
BSA	(m^2^)	2.12 ± 0.21	2.02 ± 0.17	2.19 ± 0.16	0.016
Haemoglobin	(mmol/l)	9.34 ± 0.66	9.53 ± 0.56		0.263
Rest					
HR	(beats/min)	89.5 ± 15.8	88.4 ± 15.1	79.4 ± 16.2	0.303
V’O_2_	(ml/min/kg)	5.08 ± 0.81	5.51 ± 0.90	4.57 ± 0.79	0.093
O_2_ pulse	(ml/beat)	5.24 ± 1.20	5.01 ± 0.94	5.56 ± 1.47	0.025
RER		0.84 ± 0.07	0.88 ± 0.08	0.87 ± 0.04	0.226
V’E/V’CO_2_		33.6 ± 4.4	34.4 ± 5.7	36.5 ± 3.0	0.436
SVI	(ml/m^2^)	43.4 ± 8.0	45.9 ± 6.9	41.2 ± 6.7	0.182
Cardiac index	(L/min/m^2^)	3.86 ± 0.83	4.05 ±0.96	3.31 ± 1.02	0.152
O_2_ extraction	(ml/100ml)	6.96 ± 1.76	6.70 ± 1.68	8.20 ± 2.82	0.124
Anaerobic threshold					
HR	(beats/min)	103.6 ± 14.6	107.0 ± 14.3	102.1 ± 19.2	0.529
V’O_2_	(ml/min/kg)	11.8 ± 2.8	13.4 ± 3.3	13.7 ± 3.1	0.093
O_2_ pulse	(ml/beat)	9.97 ± 2.50	9.92 ± 2.49	12.8 ± 3.5	0.025
RER		0.84 ± 0.09	0.87 ± 0.06	0.86 ± 0.08	0.250
V’E/V’CO_2_		28.0 ± 3.6	26.2 ± 3.0	27.3 ± 3.1	0.089
SVI	(ml/m^2^)	47.5 ± 8.9	51.3 ± 7.0	45.7 ± 6.4	0.049
Cardiac index	(L/min/m^2^)	4.95 ± 1.09	5.48 ± 1.07	4.77 ± 1.44	0.082
O_2_ extraction	(ml/100ml)	9.86 ± 2.02	9.74 ± 3.09	13.0 ± 4.32	0.025
Peak exercise					
HR	(beats/min)	154.6 ± 19.4	160.5 ± 20.6	151.4 ± 16.2	0.328
V’O_2_	(ml/min/kg)	24.0 ± 7.2	28.9 ± 7.1	27.3 ± 3.7	0.019
V’O_2_/pred.	%	73.9 ± 17.5	83.4 ± 19.2	96.2 ± 11.4	0.011
O_2_ pulse	(ml/beat)	13.5 ± 3.0	14.2 ± 3.0	17.1 ± 3.0	0.023
RER		1.19 ±0.11	1.24 ± 0.12	1.29 ± 0.06	0.100
V’E/V’CO_2_		28.9 ± 5.2	27.9 ± 3.8	29.2 ± 4.0	0.537
SVI	(ml/m^2^)	47.4 ± 10.5	50.4 ± 9.4	44.4 ± 8.6	0.182
Cardiac index	(L/min/m^2^)	7.44 ±2.13	8.02 ± 2.07	6.45 ±1.81	0.136
O_2_ extraction	(ml/100ml)	13.66 ± 3.31	14.63 ± 4.38	19.52 ± 6.53	0.008
Slope					
ΔQ’/ΔV’O_2_	(L/L)	5.84 ± 1.82	5.38 ± 1.63	4.01 ± 1.26	0.043

Demographic data and results of cardiopulmonary exercise tests in patients with chronic fatigue syndrome, idiopathic chronic fatigue and healthy humans during rest, at the anaerobic threshold and maximal workload. BSA: body surface area, HR: heart rate, V’O_2_: oxygen uptake, V’O_2_/pred.: oxygen uptake as percentage of predicted, SVI stroke volume index, ΔQ’/ΔV’O_2_: increase of cardiac output relative to the increase of oxygen uptake.

Post hoc Bonferroni analysis revealed that the body weight of healthy males was higher than the body weight of male CFI patients (*P* = 0.036, 95% CI: 0.70; 27.95).

Haemoglobin was not different in CFS and CFI patients (Table 1). In female patients haemoglobin was not related to O2 extraction (r = 0.077, *P* = 0.155) or to the increase of cardiac output relative to oxygen uptake (ΔQ’/ΔV’O_2_) (r = -0.008, *P* = 0.882). In male patients haemoglobin was related to O2 extraction (r = 0.245, *P* = 0.047) but not to ΔQ’/ΔV’O_2_ (r = -0.140, *P* = 0.273).

The pulmonary ventilation tests showed no difference between the CFS, CFI and healthy groups (data not shown) and the cardiac index was similar in the groups at any time during the CPET. Blood pressure was within normal limits in all tests.

At the anaerobic threshold, O_2_ extraction in healthy women was higher than in CFS (*P* = 0.008, 95% CI: 0.378; 3.273). The O_2_ extraction in healthy men was higher than in CFS (*P* = 0.044, 95% CI: 0.064; 6.221) and higher than in CFI (*P* = 0.023, 95% CI: 0.355; 6.172).

At peak exercise O_2_ extraction was higher in healthy women than in CFS (*P* = 0.010, 95% CI: 0.49; 4.74) and higher in CFI than in CFS (*P* = 0.030, 95% CI: 0.06; 1.52). The O_2_ extraction was higher in healthy men than in CFS (*P* = 0.006, 95% CI: 1.36; 10.36) and higher than in CFI (*P* = 0.018, 95% CI: 0.65; 9.13).

The lowest level of O_2_ extraction at maximal workload was 10.0 ml/100 ml in the group of healthy women, 5.4 ml/100 ml in CFI and 4.4 ml/100 ml in female CFS patients. The lowest O_2_ extraction at maximal workload in males was 12.5 ml/100 ml in the healthy group, 8.2 ml/100 ml in the CFI and 6.9 ml/100 in CFS patients.

The increase of cardiac output relative to oxygen uptake (ΔQ’/ΔV’O_2_) was lower in healthy women than CFS (*P* = 0.011, 95% CI: 0.45; 4.72) and in healthy men than CFS (*P* = 0.037, 95% CI: 0.08; 3.58).

In the fatigue patients a low oxygen extraction (≤10 ml/100 ml) coincided with a increased response time of 2.35?±?0.19 versus 1.79?±?0.13 (*P* = 0.001, 95% CI: -1.19; -0.33) in the Shifting Attentional test visual of the ANT.

## Discussion

The lower maximal exercise capacity (peak V’O_2_) of CFS and CFI patients was related to a lower oxygen uptake of the muscle cells (C(a-v)O_2_) and a higher increase of cardiac output relative to V’O_2_ (ΔQ’/ΔV’O_2_) than in healthy men and women.

The lower peak V’O_2_ was not explained by an impairment of ventilation. The stroke volume and cardiac output increased during the exercise test, but at no level of effort a consistent difference was seen between the three groups, indicating a normal adaptation of the heart to increasing workload in CFS patients. This result was in accordance with previous studies [2,4,19]. Stroke volume in men (n = 83) during exercise test was not different from reported values by Nexfin [17], gas rebreathing [5] and impedance cardiography [20]. Resting values were 78.9?±?12.8 ml in this study, 80?±?9 ml by Nexfin and 73.8?±?10.1 ml by impedance cardiography and at peak 100.7?±?18.0 ml in this study, 107.5?±?7.2 ml by gas rebreathing and 97.9?±?6.4 ml by impedance cardiography. Peak cardiac output in men was 192.0?±?92.9 ml/kg/min in this study and 212?±?37 ml/kg/min by acetylene rebreathing [6].

We have no haemoglobin data of the healthy visitors. It is possible that the haemoglobin values of patients, although within normal limits [21], were lower than the values of healthy visitors. The mean value of the healthy female visitors would need to be ±10 mmol/l (1 mmol Hb?≈?1.5 ml/100 ml O2 extraction) to explain the difference of O2 extraction by a difference of haemoglobin [15]. A high cardiac index, caused by low haemoglobin would also have been present during rest [22], but we found no difference between the 3 groups. In men haemoglobin correlated with oxygen extraction, but explained only 6% of the variance at peak V’O2.

The oxygen extraction increases during incremental exercise and a lower value of peak oxygen extraction in the CFS and CFI groups might be attributed to insufficient effort, caused by lack of motivation. This, however, would not explain the lower oxygen extraction at the anaerobic threshold and the higher slope of the ΔQ’/ΔV’O_2._ Another cause for the lower V’O_2_ in CFS and CFI could be the deconditioning in these patients, but the value of the increase of cardiac output relative to the oxygen uptake (ΔQ’/ΔV’O_2_) is independent from motivation and deconditioning [6] (normal ΔQ’/ΔV’O_2_?≈?5).

The most probable cause for the low peak V’O_2_, the low oxygen extraction and the high ΔQ’/ΔV’O_2_ in CFS and CFI patients was an attenuated cell metabolism. The low oxygen extraction during exercise was also reported in mitochondrial pathology [6], systemic lupus erythematosus [23], HIV [5] and myophosphorylase deficiency [24].

This result is also not in contradiction to the abnormal proton handling that was reported during and after cessation of exercise in CFS patients [25,26].

**Figure 1:**
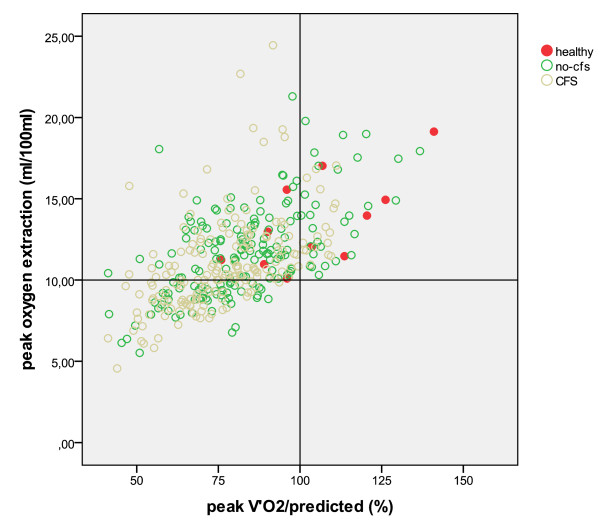
**The peak oxygen uptake as percentage of predicted relative to the peak oxygen extraction.** Vertical line: predicted peak oxygen uptake. Horizontal line: lowest oxygen extraction reported for healthy humans. No-CFS: Idiopathic Chronic Fatigue, CFS Chronic Fatigue Syndrome.

The peak oxygen extraction in men and women who performed at the same level or better than the reference population of sedentary healthy subjects [27] was never less than 10 ml/100 ml as reported for healthy male subjects [28] (Figure 1). The same lower limit of 10 ml/100 ml was also reported in heart failure patients [29]. The O_2_ extraction of healthy participants in this study was higher than 10 ml/100 ml (Figure 1). The mean O_2_ extraction at maximal workload in fatigue patients was much lower and comparable to asymptomatic HIV infected individuals (10.8?±?0.5 ml/100 ml) [5].

The peak V’O_2_ of 73 CFS patients and 59 CFI patients was the same as or higher than the mean peak V’O_2_ of healthy sedentary people [27]. All CFS and CFI patients, however, experienced a physical impairment that was severe enough for the diagnosis. The conclusion must be that the subjective experience of physical impairment and the objective peak V’O_2_ in the CPET are not identical.

If the mitochondrial system is intact in CFS patients [4], the low oxygen extraction in a subgroup of CFS patients may indicate a downregulation of the activity in vivo. A downregulation by a factor that is involved in the activity of the immune system would explain the same phenomenon in SLE [23], different from damaged mitochondria in HIV [5,30]. The lower oxygen extraction and higher ΔQ’/ΔV’O_2_ however do not differentiate between down regulation of cell metabolism and congenital or acquired mitochondrial pathology in CFS patients. The abnormal results of the Shifting Attentional test visual of the ANT suggest that the impaired oxygen uptake is not limited to the muscle cells.

### Limitations

The validity of the results of this retrospective observational study is limited by the uncontrolled inclusion of the participants. The exercise protocol for healthy visitors was not different from the protocol for fatigued patients but we have no laboratory data of healthy visitors. Therefore we cannot exclude less than optimal results in this group due to unknown diseases. The accuracy and precision of the assessment of stroke volume by Nexfin in CFS patients during CPET was not reported yet. For accuracy we used the data of comparable studies in healthy subjects, the results of a study of repeated CPET’s is needed for the assessment of precision. We cannot exclude influence of haemoglobin on the oxygen transport capacity of the blood in healthy visitors.

## Conclusions

CPET with continuous measuring of cardiac output by Nexfin allowed for the calculation of the presence and severity of metabolic causes of exercise intolerance. This retrospective study showed that a low oxygen extraction and a high ΔQ’/ΔV’O_2_ were consistent with a metabolic cause for exercise intolerance in 70% of CFS patients.

## Competing interests

The authors declare that they do not have competing interests.

## Authors’ contributions

RV and IV contributed to the collection and analysis of the data. The first drafts of the paper were written by RV and RV and IV contributed to the final version of the article. Both authors read and approved the final manuscript.
